# Achilles Tendon Xanthoma Inflammation Revealed by FDG-PET/CT in Familial Hypercholesterolemia: Insights From a 2-Generation Case Series

**DOI:** 10.1016/j.jaccas.2026.106949

**Published:** 2026-02-12

**Authors:** Takaharu Nakayoshi, Nobuhiro Tahara, Yayoi Sakata, Shuichi Tanoue, Yoshihiro Fukumoto

**Affiliations:** a Division of Cardiovascular Medicine, Department of Medicine, Kurume, Japan; b Center for Diagnostic Imaging, Kurume, Japan; c Department of Radiology, Kurume University School of Medicine, Kurume, Japan

**Keywords:** Achilles tendon xanthoma, familial hypercholesterolemia, FDG-PET/CT, inflammation, lipid-lowering therapy

## Abstract

**Background:**

Achilles tendon xanthomas are hallmark lesions of familial hypercholesterolemia (FH), but their metabolic activity remains poorly characterized.

**Case Summary:**

We report a 2-generation case series illustrating phenotypic heterogeneity in tendon xanthomas despite shared FH-associated variants. In case 1, a 35-year-old man with bilateral Achilles tendon thickening was diagnosed with FH. After 12 months of lipid-lowering therapy, intense ^18^F-fluorodeoxyglucose (FDG) uptake on positron emission tomography/computed tomography (PET/CT) indicated metabolically active inflammation. In case 2, the man's 71-year-old father, who had been receiving long-term statins (>10 years), ezetimibe (>1 year), and recent proprotein convertase subtilisin/kexin type 9 inhibitor (1 month), had Achilles tendon thickening but no FDG uptake despite age and coronary artery disease history.

**Discussion:**

These findings show that xanthomas exhibit persistent metabolic activity despite short-term lipid lowering, whereas prolonged therapy may attenuate inflammation. Phenotypic differences underscore roles of disease stage, cumulative lipid exposure, and treatment duration. FDG-PET/CT provides metabolic information for risk assessment and therapeutic monitoring.

## Visual Summary

**Figure:**
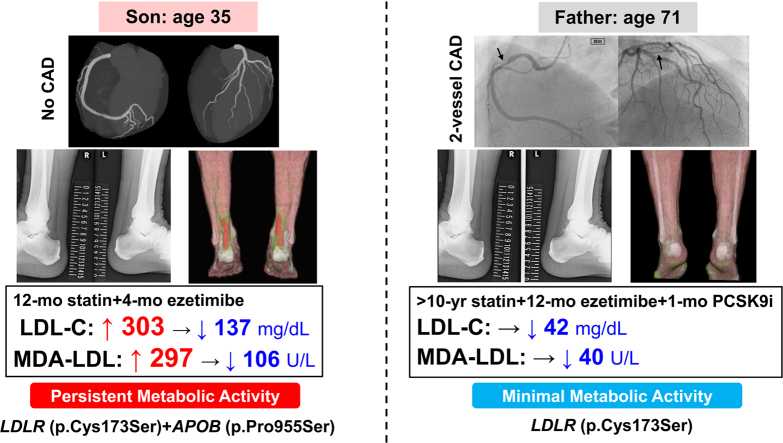

## Case 1: 35-Year-Old Son

### Presentation and clinical findings

A 35-year-old man with untreated low-density lipoprotein (LDL) cholesterol of 303 mg/dL (reference <140 mg/dL) was referred for atherosclerotic cardiovascular disease (ASCVD) risk assessment. He had no cardiovascular history or symptoms. Physical examination revealed bilateral Achilles tendon thickening without xanthelasma or corneal arcus.

**Take-Home Messages**

• FDG-PET/CT identifies metabolically active Achilles tendon xanthomas, providing metabolic information beyond structural imaging in familial hypercholesterolemia.
• Metabolic activity in tendon xanthomas may persist despite short-term intensive lipid-lowering therapy, suggesting that longer or more aggressive treatment may be required to achieve metabolic quiescence.
• Phenotypic heterogeneity exists even within families, highlighting the value of integrating molecular imaging with genetic and biochemical profiling to guide individualized risk assessment and management.

### Diagnostic assessment

**Figure 1:**
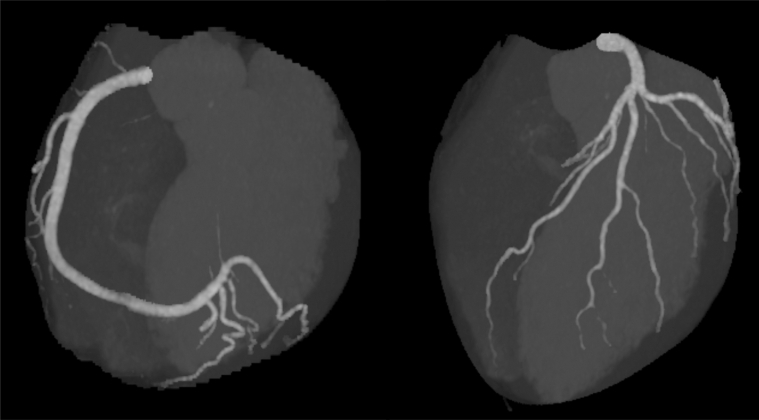
Coronary CT Angiography in the 35-Year-Old Son Coronary CT angiography demonstrating normal coronary anatomy without stenosis or plaque and a calcium score of 0. CT = computed tomography.

**Figure 2:**
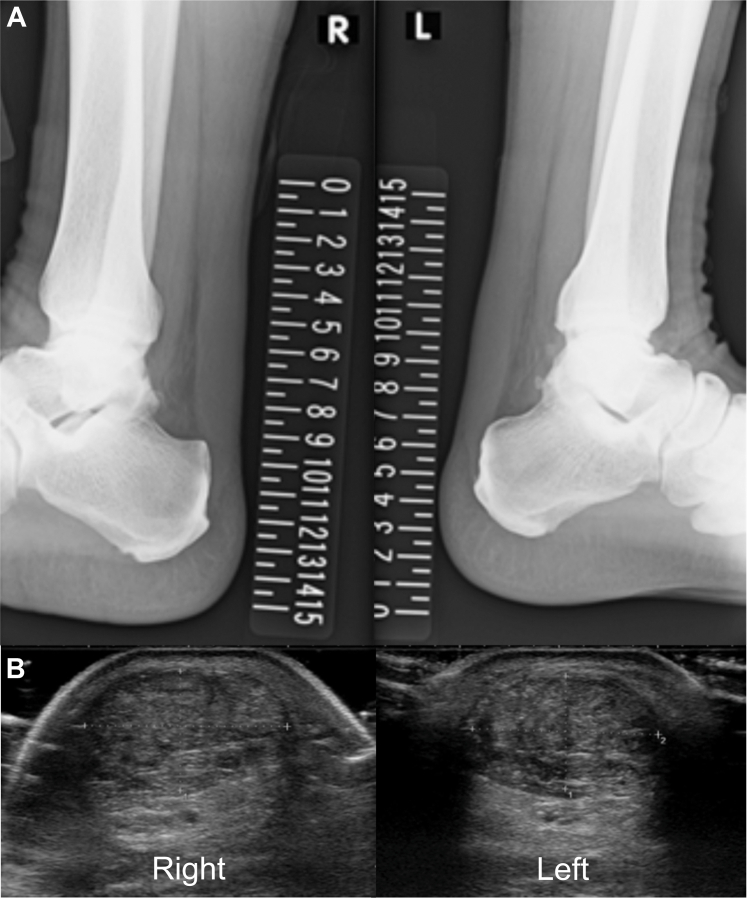
Structural Imaging of Achilles Tendons in the 35-Year-Old Son (A) Lateral ankle radiography showing increased soft tissue density consistent with Achilles tendon thickening. (B) Ultrasonography revealing marked bilateral thickening (right: 11.8 mm, left: 11.2 mm), heterogeneous echotexture, and hyperechoic foci.

Laboratory findings demonstrated markedly elevated malondialdehyde-modified LDL (MDA-LDL: 297 U/L, reference: 61-105 U/L) and mildly increased remnant-like particle (RLP) cholesterol (10.2 mg/dL, reference: <7.5 mg/dL), whereas lipoprotein(a) (9 mg/dL, reference: <30 mg/dL), triglycerides (90 mg/dL), and high-density lipoprotein cholesterol (56 mg/dL) were within normal limits. Electrocardiography, echocardiography, and exercise stress testing yielded unremarkable findings. Coronary computed tomography (CT) angiography demonstrated no stenosis or plaque, with a calcium score of 0 (Figure 1). Plain radiography showed increased Achilles soft tissue density (Figure 2A). Ultrasonography confirmed bilateral tendon thickening (right: 11.8 mm, left: 11.2 mm) at 2 cm proximal to the calcaneal insertion, with heterogeneous echotexture and hyperechoic foci (Figure 2B). Genetic testing identified compound heterozygous pathogenic variants: c.517T>A (p.Cys173Ser) in *LDLR* (classified as pathogenic by American College of Medical Genetics and Genomics [ACMG]/ClinGen criteria) and c.2863C>T (p.Pro955Ser) in *APOB* (classified as pathogenic/likely pathogenic by ACMG/ClinGen criteria). Based on the Dutch Lipid Clinic Network criteria (score: 10 points), a diagnosis of definite familial hypercholesterolemia (FH) was established.

### Management

High-intensity statin therapy with rosuvastatin was initiated and was titrated to 20 mg daily. Ezetimibe (10 mg) was added 8 months later owing to suboptimal LDL cholesterol reduction.

### Follow-up and outcome

**Figure 3:**
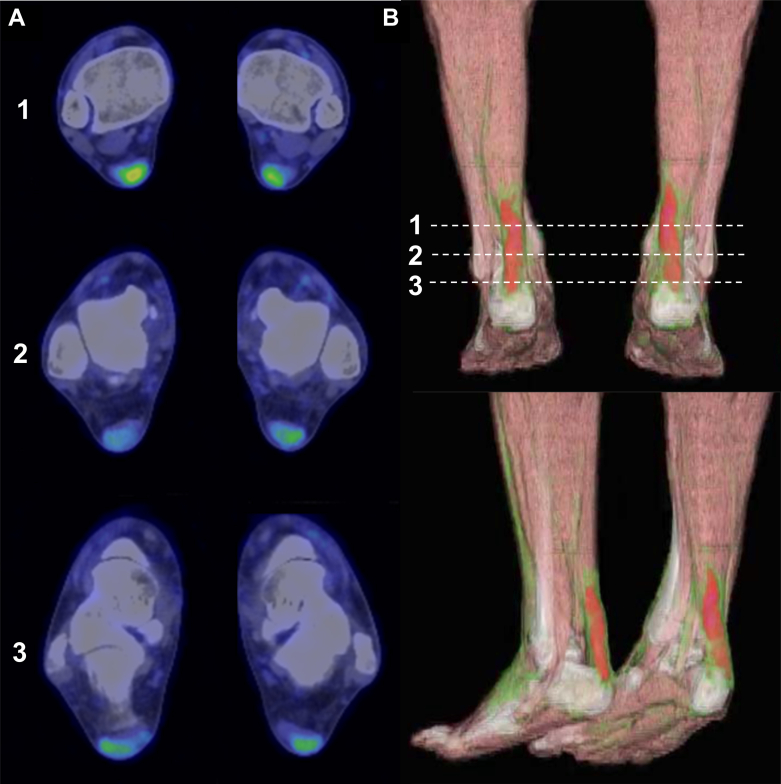
Metabolic Activity of Achilles Xanthomas on FDG-PET/CT in the Son FDG-PET/CT showing intense bilateral tracer uptake within the Achilles tendons, consistent with active inflammatory xanthomas. FDG-PET/CT = ^18^F-fluorodeoxyglucose positron emission tomography/computed tomography.

After 12 months of lipid-lowering therapy (4 months after ezetimibe addition), ^18^F-fluorodeoxyglucose positron emission tomography/computed tomography (FDG-PET/CT) demonstrated intense uptake in both Achilles tendons, indicating metabolically active lesions that were highly suggestive of inflammation (Figure 3, Video 1). At that time, LDL cholesterol had decreased to 137 mg/dL, with MDA-LDL and RLP cholesterol reduced to 106 U/L and 2.1 mg/dL, respectively. Notably, persistent FDG activity despite intensive lipid-lowering therapy suggested ongoing inflammatory activity within the xanthomas.

## Case 2: 71-Year-Old Father

### Presentation and clinical history

**Figure 4:**
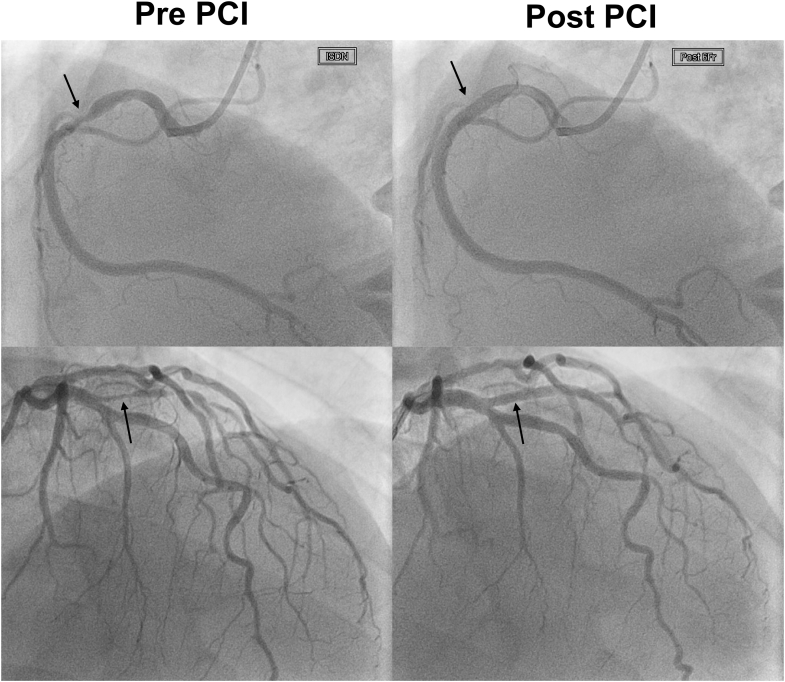
Coronary Angiography in the 71-Year-Old Father Coronary angiography demonstrating prior percutaneous coronary intervention (PCI) with drug-eluting stents placed in the right coronary artery and intermediate branch (arrows).

The first patient's 71-year-old father, diagnosed with FH at age 68, had undergone percutaneous coronary intervention for stable angina in his 60s with drug-eluting stents placed in the right coronary artery and intermediate branch (Figure 4, arrows). A strong familial clustering of premature coronary artery disease was evident, as his father and multiple siblings had experienced myocardial infarction in their 50s.

### Diagnostic assessment

**Figure 5:**
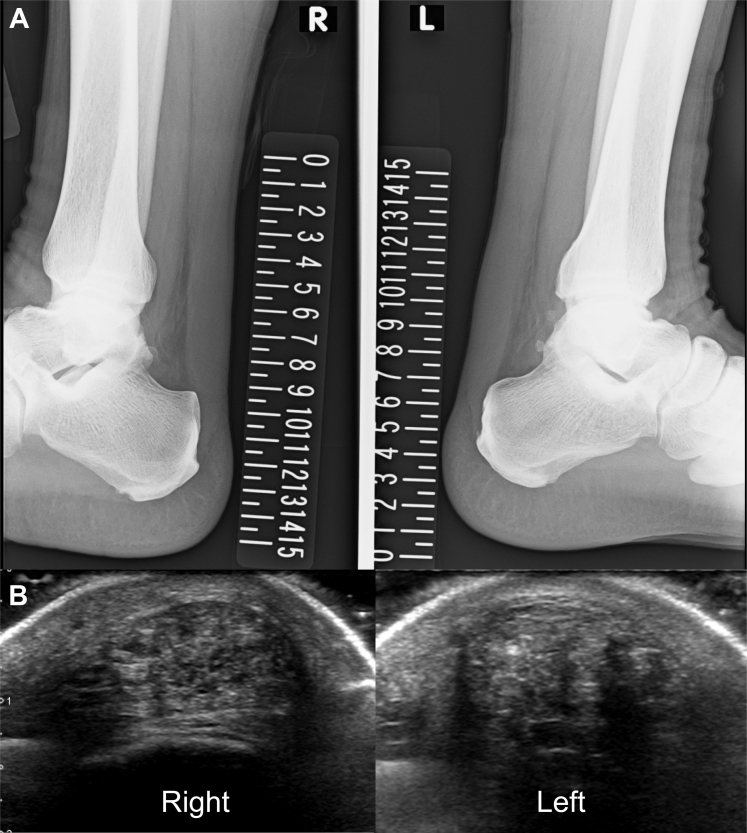
Structural Imaging of Achilles Tendons in the 71-Year-Old Father (A) Lateral ankle radiography showing bilateral Achilles tendon thickening with microcalcifications. (B) Ultrasonography demonstrating moderate thickening (right: 8.3 mm, left: 9.5 mm) and heterogeneous echotexture.

Genetic testing revealed heterozygosity for c.517T>A (p.Cys173Ser) (classified as pathogenic by ACMG/ClinGen criteria) in *LDLR*. Similar to his son, bilateral Achilles tendon thickening with microcalcifications was observed on radiography (Figure 5A). Ultrasonography confirmed tendon thickness of 8.3 mm (right) and 9.5 mm (left) with heterogeneous echotexture (Figure 5B), indicating milder structural changes compared with his son.

### Treatment and imaging findings

**Figure 6:**
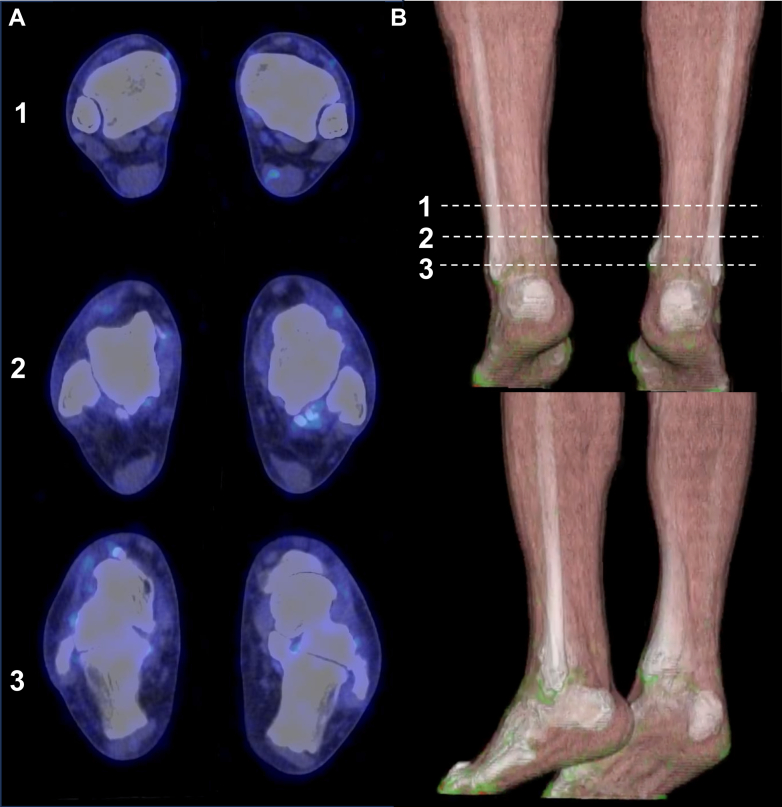
FDG-PET/CT of Achilles Tendons in the 71-Year-Old Father FDG-PET/CT showing no significant tracer uptake in either Achilles tendon, indicating absence of detectable inflammatory activity. FDG-PET/CT = ^18^F-fluorodeoxyglucose positron emission tomography/computed tomography.

He had been on rosuvastatin 10 mg daily for over a decade, with ezetimibe 10 mg added more than 1 year prior. Evolocumab 420 mg subcutaneously monthly was initiated 1 month before imaging. FDG-PET/CT demonstrated no significant tracer uptake in either Achilles tendon (Figure 6, Video 2). At the time of imaging, atherogenic lipid parameters were markedly reduced to levels well below reference ranges (LDL cholesterol: 42 mg/dL, MDA-LDL: 40 U/L, and RLP cholesterol: 1.5 mg/dL), whereas high-density lipoprotein cholesterol (50 mg/dL), triglycerides (61 mg/dL), and lipoprotein (a) (8 mg/dL) were within reference ranges.

## Discussion

Achilles tendon xanthomas are observed in up to 50% of patients with FH, with prevalence influenced by age, genotype, and cumulative LDL cholesterol exposure.^1^ These lesions result from extracellular cholesterol deposition that triggers chronic inflammatory responses including macrophage infiltration, foam cell formation, and extracellular matrix remodeling. While ultrasound and radiography effectively delineate structural abnormalities such as tendon thickening and calcifications,^2^ these modalities cannot assess the underlying inflammatory activity within xanthomas. FDG-PET/CT uniquely visualizes cellular metabolic activity, which predominantly reflects glucose consumption by inflammatory cells, and it has emerged as a promising modality for exploring xanthoma pathobiology and its link to metabolic activity in ASCVD.^3^ The ability to detect increased glucose metabolism in inflammatory cells may have implications for risk stratification and monitoring treatment response in FH.

### Phenotypic divergence in a shared genetic background

This case series illustrates striking intrafamilial divergence in FDG-PET/CT findings despite a shared genetic background. The younger patient harboring compound heterozygous variants in *LDLR* (p.Cys173Ser) and *APOB* (p.Pro955Ser) demonstrated intense FDG activity in both Achilles tendons despite the complete absence of coronary atherosclerosis on CT angiography and a calcium score of 0. This dissociation between increased tendon metabolic activity and vascular involvement suggests that glucose-avid inflammatory cells in xanthomas may accumulate prior to or independently of overt ASCVD, raising the possibility that metabolically active tendon xanthomas could serve as an early marker of systemic inflammatory dysregulation in FH. In contrast, his father carried the *LDLR* variant (p.Cys173Ser) without an *APOB* variant and had coronary artery disease requiring prior percutaneous coronary intervention. Despite bilateral structural abnormalities, no tendon FDG activity was detected. Notably, his Achilles tendons were slightly thinner than his son's (8.3-9.5 mm vs 11.2-11.8 mm), and imaging revealed predominantly calcified rather than soft tissue components. The phenotypic discordance within this family is striking; the son showed xanthomas with increased glucose metabolism despite the absence of coronary disease, whereas the father exhibited xanthomas without detectable metabolic activity despite advanced atherosclerosis. This contrast underscores the complex and heterogeneous nature of FH manifestations even within a single family.

### Potential mechanisms underlying phenotypic discordance

#### Genotypic differences

The son's additional *APOB* (p.Pro955Ser) variant, affecting the ligand-binding domain of apolipoprotein B-100 (apoB-100), may contribute to his inflammatory phenotype. This variant impairs LDL receptor recognition, potentially leading to prolonged circulatory time of atherogenic lipoproteins and enhanced oxidative modification.^4^ Oxidized LDL particles are potent chemoattractants for monocytes and stimulate macrophage activation and foam cell formation within xanthomas. The markedly elevated MDA-LDL level in the son (297 U/L at baseline, 106 U/L after treatment) supports ongoing oxidative stress and may reflect heightened local inflammatory responses within tendon tissues. In contrast, the father's genotype restricted to *LDLR* variant without *APOB* involvement may underlie a distinct pattern of lipoprotein metabolism and tissue deposition, although the mechanistic basis of this difference remains speculative.

#### Disease stage and inflammatory activity

The natural history of xanthomas likely involves distinct metabolic phases. In the son's case, intense FDG avidity with relatively homogeneous soft tissue density suggests the presence of metabolically active cells within the xanthomas. Conversely, the father's xanthomas characterized by prominent calcifications and absent FDG activity may reflect a composition dominated by fibrotic and calcific elements with fewer metabolically active cells. Whether this difference reflects true disease stage progression, inherent biological heterogeneity, or the effects of prolonged treatment remains unclear.

#### Duration and intensity of lipid-lowering therapy

The son's persistent FDG activity despite 12 months of treatment, including progressive high-intensity statin titration and 4 months of combination therapy, suggests that glucose metabolism in xanthoma inflammatory cells may be relatively resistant to short-term intervention. The residual elevation of oxidative stress markers (MDA-LDL: 106 U/L) despite aggressive therapy demonstrates that reduction of inflammatory cell metabolic activity requires not only adequate dosing but also sufficient treatment duration, though longer follow-up is needed to confirm whether metabolic activity eventually subsides in this patient. In contrast, the father's complete absence of FDG activity after more than 2 years of intensive dual lipid-lowering therapy and marked suppression of MDA-LDL to 40 U/L after initiation of a PCSK9 inhibitor suggests that sustained, profound lipid control may contribute to inflammation reduction and metabolic quiescence in tendon xanthomas. However, given the cross-sectional nature of this observation and the differences in disease stage between father and son, we cannot definitively attribute the father's lack of FDG activity solely to treatment duration rather than to inherent differences in xanthoma biology or inflammatory phase.

#### Imaging considerations

Several technical factors warrant consideration when interpreting FDG-PET/CT findings in Achilles tendon xanthomas. Partial-volume effects inherent to PET imaging may underestimate FDG activity in small or fibrotic structures, potentially obscuring true metabolic activity. However, both patients' Achilles tendons exceeded 8 mm in thickness, within an acceptable range relative to the typical spatial resolution of clinical PET systems. The temporal dynamics of xanthoma inflammation may also affect FDG activity. Active macrophage infiltration and foam cell formation, characteristic of early xanthoma development, are metabolically demanding and manifest as increased FDG activity. In contrast, mature xanthomas with progressive fibrosis and calcification may exhibit reduced cellular density and metabolic activity despite persistent structural abnormality. In the father's case, the complete absence of FDG activity could theoretically reflect several scenarios: 1) xanthomas with minimal metabolically active inflammatory cells from the outset; 2) reduction of inflammatory cell glucose metabolism achieved through prolonged intensive lipid-lowering therapy; or 3) progression to a predominantly fibrotic stage with few glucose-consuming cells. While his profound lipid control with near-complete normalization of atherogenic lipoproteins (LDL cholesterol: 42 mg/dL, MDA-LDL: 40 U/L) and the striking contrast with his son's intense activity under identical imaging conditions argue against a purely technical limitation, the cross-sectional nature of this observation precludes definitive determination of whether the absence of metabolic activity represents treatment effect or inherent xanthoma biology.

#### Clinical implications

From a translational perspective, these observations underscore the complementary role of FDG-PET/CT alongside anatomic imaging in FH. While structural imaging remains the standard for xanthoma detection, FDG-PET/CT provides unique metabolic information that may refine risk assessment and guide therapeutic strategies. Detection of increased glucose metabolism in tendon xanthomas in a young, asymptomatic patient without coronary involvement (case 1) may identify a high-risk phenotype characterized by metabolically active inflammatory cells that warrants intensified preventive strategies such as earlier consideration of PCSK9 inhibitor therapy or more frequent cardiovascular surveillance. The persistent FDG activity despite 1 year of intensive therapy highlights the point that short-term lipid lowering may not immediately suppress inflammatory cell metabolic activity in xanthomas, suggesting the need for sustained treatment and potentially longer follow-up periods before metabolic quiescence is achieved. Conversely, the absence of FDG activity in the well-treated father (case 2) despite longstanding structural changes suggests that prolonged aggressive lipid control may favorably alter the inflammatory course of tendon lesions and achieve metabolic quiescence, though this cannot be definitively confirmed without baseline imaging. This finding provides reassurance that intensive therapy has achieved not only biochemical but also biological control of xanthoma inflammation. Whether tendon FDG activity independently predicts ASCVD events, reflects treatment responsiveness, or simply marks a transient inflammatory phase remains to be determined through longitudinal studies. The detection of this inflammation provides complementary metabolic insight for individualized risk assessment and treatment guidance that is not obtainable through conventional structural or genetic profiling alone. Currently, cost, radiation exposure, and limited accessibility preclude widespread clinical application of FDG-PET/CT in FH management. However, in selected cases where treatment optimization is uncertain or cardiovascular risk stratification is challenging, FDG-PET/CT may offer valuable adjunctive information.

**Visual Summary:**
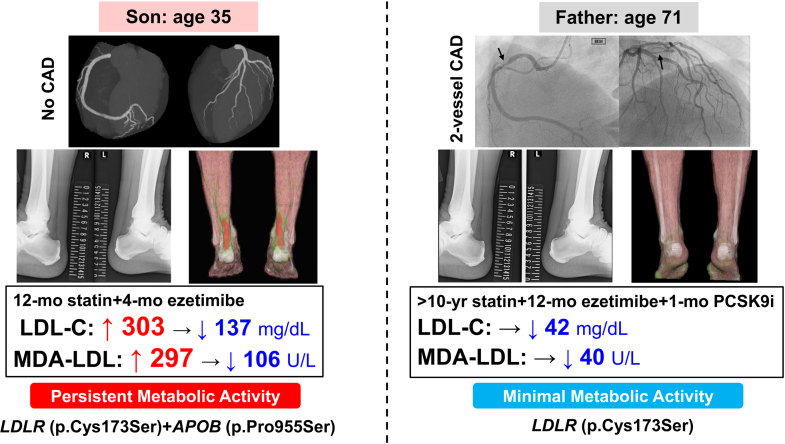
Differential Achilles Tendon Xanthoma Metabolic Activity: Impact of Genotype and Treatment Duration CAD = coronary artery disease; FDG-PET/CT = ^18^F-fluorodeoxyglucose positron emission tomography/computed tomography; LDL-C = low-density lipoprotein cholesterol; MDA = malondialdehyde-modified.

## Conclusions

This father-son comparison underscores the biological heterogeneity of FH that extends beyond genetic classification alone. The discordant inflammatory profiles, despite partial genotypic overlap, highlight the potential of integrating molecular imaging with genetic and biochemical profiling to refine phenotypic characterization and personalize risk assessment. Longitudinal studies correlating changes in tendon FDG activity with treatment duration, systemic inflammatory markers, and cardiovascular outcomes are warranted to determine whether imaging-detected inflammation represents a modifiable therapeutic target in FH.

## Funding Support and Author Disclosures

The authors have reported that they have no relationships relevant to the contents of this paper to disclose.

## Appendix

Video 1 FDG-PET/CT Fusion Image of the 35-Year-Old Son With Familial Hypercholesterolemia Intense FDG activity is observed along both Achilles tendons, highlighting metabolically active inflammatory xanthomas not discernible on anatomic imaging alone.

Video 2 FDG-PET/CT Fusion Image of the 71-Year-Old Father With Familial Hypercholesterolemia Despite bilateral Achilles tendon thickening and calcifications on radiography and ultrasonography, no significant FDG activity is observed, consistent with metabolically inactive xanthomas.
